# Efficacy of Pirikool® 300 CS used for indoor residual spraying on three different substrates in semi-field experimental conditions

**DOI:** 10.1186/s12936-024-04912-3

**Published:** 2024-05-15

**Authors:** Behi Kouadio Fodjo, Emile Tchicaya, Laurence Aya Yao, Constant Edi, Alassane Foungoye Ouattara, Loukou Bernard Kouassi, Firmain N’dri Yokoly, Koudou Guibéhi Benjamin

**Affiliations:** 1 Centre Suisse de Recherches Scientifiques en Côte d’Ivoire (CSRS), Abidjan, Côte d’Ivoire; 2 Université Nangui Abrogoua, Abidjan, Côte d’Ivoire; 3 Université Peleforo Gon Coulibaly (UPGC), Korhogo, Côte d’Ivoire

**Keywords:** Malaria, *Anopheles gambiae*, Resistance, Pirikool® 300CS, Indoor residual spraying, Experimental huts

## Abstract

**Background:**

Vector control using insecticides is a key prevention strategy against malaria. Unfortunately, insecticide resistance in mosquitoes threatens all progress in malaria control. In the perspective of managing this resistance, new insecticide formulations are being tested to improve the effectiveness of vector control tools.

**Methods:**

The efficacy and residual activity of Pirikool® 300 CS was evaluated in comparison with Actellic® 300 CS in experimental huts at the Tiassalé experimental station on three substrates including cement, wood and mud. The mortality, blood-feeding inhibition, exiting behaviour and deterrency of free-flying wild mosquitoes was evaluated. Cone bioassay tests with susceptible and resistant mosquito strains were conducted in the huts to determine residual efficacy.

**Results:**

A total of 20,505 mosquitoes of which 10,979 (53%) wild female *Anopheles gambiae* were collected for 112 nights. Residual efficacy obtained from monthly cone bioassay was higher than 80% with the susceptible, laboratory-maintained *An. gambiae* Kisumu strain, from the first to the tenth study period on all three types of treated substrate for both Actellic® 300CS and Pirikool® 300CS. This residual efficacy on the wild Tiassalé strain was over 80% until the 4th month of study on Pirikool® 300CS S treated substrates. Overall 24-h mortalities of wild free-flying *An. gambiae *sensu lato which entered in the experimental huts over the 8-months trial on Pirikool® 300CS treatment was 50.5%, 75.9% and 52.7%, respectively, on cement wall, wood wall and mud wall. The positive reference product Actellic® 300CS treatment induced mortalities of 42.0%, 51.8% and 41.8% on cement wall, wood wall and mud wall.

**Conclusion:**

Pirikool® 300CS has performed really well against resistant strains of *An. gambiae* using indoor residual spraying method in experimental huts. It could be an alternative product for indoor residual spraying in response to the vectors' resistance to insecticides.

## Background

Vector control is a key component of the worldwide malaria control strategy [1, 2]. It helps to reduce malaria incidence and saves the lives of around 220,000 African children under the age of five every year [3, 4]. Vector control is the most recommended option of prevention against several vector-borne diseases for which vaccinations are not currently available [5]. Collective vector control methods are mainly long-lasting insecticidal nets (LLINs) and indoor residual spraying (IRS). Although IRS has declined in endemic countries in recent years, it is the second most implemented vector control intervention by national malaria control programmes [2] and has protected about 80 million people worldwide in 2022 [2]. IRS's efficiency has been demonstrated by its active contribution to malaria eradication in some European and Asian nations including in South Africa [6]. IRS has also helped to reduce malaria in African nations where they are widely used [7, 8].

For many years, only four insecticides classes (carbamates, organochlorines, organophosphates, or pyrethroids) were used for IRS, resulting in overuse of the same chemicals and a significant selection pressure for insecticide resistance. Insecticide resistance is increasing in malaria vectors, jeopardizing the efficiency of vector control methods and thereby undoing recent success in malaria management. One response to this problem has been the diversification of insecticide formulations with residual effects [9].

Thus, novel products combining insecticide combinations, new formulations of current insecticides, or reformulation of some insecticides traditionally used solely in agriculture have been produced [10–14, 14, 15]. Some of these insecticides have previously been shown to be effective in real-world contexts where malaria transmission is prevalent. [15–17]. However, in order to prevent the development of resistance, it is critical to diversity the number of compounds utilized. However, in order to prevent the development of resistance, it is critical to diversity the number of compounds utilized.

In this context, Tianjin Yorkool International Trading Co, Ltd. has developed a new micro-encapsulated formulation (CS) of Pirimiphos-methyl called Pirikool® 300 CS that has a longer lasting effectiveness. This compound showed good performance in indoor residual spraying in experimental huts in Benin against pyrethroid-resistant vectors [18]. However, in some localities, such as Tiassalé in Côte d'Ivoire, malaria vector resistance is extended to all classes of public health insecticides [19–21]. Because vector control treatments are typically used in a variety of epidemiological contexts, it is necessary to assess the efficiency of any new product on such a mosquito strain as well. In light of this, a research project was launched in Tiassalé to further evaluate the efficiency of Pirikool® 300 CS against multi-insecticide resistant mosquito strains. Furthermore, as part of the WHO Prequalification Team's (PQT-VCP) process of prequalifying vector control products, the number, Type, and duration of trials, as well as the selection of assessment locations, are part of the WHO criteria that establish the quality of any new product [22].

## Methods

### Study site

The research was carried out at Tiassalé (5°54ʹ N. 4°50ʹ W), in Southern Côte d'Ivoire, in a rice irrigated field. The region's principal malaria vector is a member of the *Anopheles gambiae* complex, *Anopheles coluzzii*, which is resistant to pyrethroids, organochlorine (DDT), carbamates, and organophosphates. Knock down resistance (*kdr*) and *ace-1R* gene frequencies are extremely high, with values of 0.8 and 0.44, respectively. The N1575Y mutation was also found in this group. In Tiassalé, metabolic resistance mediated by cytochrome P450 genes (CYP6 family), carboxylesterases, and glutathione S-transferases was linked to insecticide resistance [21, 23].

### Experimental hut design

The study included 21 West-African-style experimental huts [24]. The design is based on traditional local dwelling, with significant adaptations for mosquito entry and escape control [25]. There were three types of huts used: concrete brick, mud, and wooden, all with a corrugated iron roof and ceiling. Each hut is built on a concrete foundation and is encircled by a water pipe to keep ants away from the dead mosquitoes. Mosquitoes enter the hut via four modified 2 cm wide apertures on three sides (front and two sides). Mosquitoes can escape through a little veranda on the fourth side [26].

### Study design

For each type of construction, three huts were used per treatment arm and the controls respectively to make a total of 21 huts for the trial according to the following:

#### Candidate product

Three wooden huts treated with Pirikool® 300CS (1000 mg AI/m^2^).

Three mud huts treated with Pirikool® 300CS (1000 mg AI/m^2^).

Three cement (concrete brick) huts treated with Pirikool® 300CS (1000 mg AI/m^2^).

#### Positive control

Three wooden huts treated with Actellic® 300CS (1000 mg AI/m^2^).

Three mud huts treated with Actellic® 300CS (1000 mg AI/m^2^).

Three cement huts treated with Actellic® 300CS (1000 mg AI/m^2^).

#### Negative control

Three concrete brick control cement huts treated with water used as negative control.

### Experimental hut treatments

The walls and ceiling of each experimental hut were sprayed using a Hudson X-pert compression sprayer. The application rate was 1000 mg/m^2^ for each insecticide (Actellic® 300CS and Pirikool® 300CS). To make things simpler, a 75 cm (5 cm overlapping) band was written on the wall with white chalk, and the 4.5 s was respected for each band, as well as a guiding pole connected to the end of the spray lance to keep a set distance to the wall.

### Mosquito collections and proceedings

Monthly collections were done every two weeks for eight months. Sleepers entered the hut about 8 p.m. and stayed until 6 a.m. Mosquitoes are collected daily, between the hours of 6 and 8 a.m. Sleepers in the hut slept with untreated holes bed nets. Mosquito collections were made individually from the veranda, room, and net, with accurate records of location kept and entered in the record datasheet. Haemolysis tubes were used to gather resting and dead mosquitoes from inside the net, the room, and the veranda traps. Mosquitoes from each of these collection sites were identified to Genus and, to the extent possible in the field, to species level (i.e. *Anopheles gambiae *sensu lato, which will be referred to as *An. gambiae* from now on in the text). They were graded as dead/alive, unfed/blood fed, half gravid/gravid. To test 24-h delayed mortality, live mosquitoes are put in cups and exposed to a 10% sugar solution for one day. The temperature ranged from 23 to 27 °C, with a relative humidity of 70–80%. The data was reported on daily record sheets and double-entered into an Excel spreadsheet.

### Cone bioassays

Cone testing was used to determine the residual effectiveness of the insecticide on the treated surfaces one week after spraying and then monthly for ten months. Twenty cones were set on the wall at various heights (0.5, 1.0, and 1.5 m above ground) and on the sprayed ceiling into the sprayed huts. Ten susceptible reference Kisumu *An. gambiae* females were placed into each cone and subjected for 30 min. After 60 min, the knockdown was documented, and death was reported 24 h later.

### Chemical analysis

Prior to spraying, six filter-papers (5 × 5 cm Whatman 1) were taped at different points on the walls to test spray quality. After spraying, they were allowed to dry for 24 h before being covered in aluminum foil and kept at 4° C until HPLC analysis at Vaster Testing Technology Co..Ltd.

### Outcome measures

The outcome metrics of mortality, induced mortality, deterrence, and personal protection were used to assess the efficacy of the treatments. The number of dead *An. gambiae* at each timepoint was divided by the total number of *An. gambiae* captured to determine mortality. The mean percentage mortality of *An. gambiae* caught per treatment was displayed with a 95% confidence interval. For Blood Fed, a variable was created for fed mosquitoes by dividing the amount collected by the sum of fed living and fed dead mosquitos. The mean percentage of fed living *An. gambiae* mosquitoes collected per treatment with a 95% confidence interval was then determined. For Induced Exophily, a variable was constructed for exophily that was stated as a percentage of the total number of mosquitoes discovered in the veranda trap over the total number of mosquitoes found in the hut and in the veranda trap. The mean percentage of *An. gambiae* exophily per treatment was presented, along with the 95% CI. Deterrence outcomes were calculated by subtracting the total caught in untreated arms from the total captured in treated arms and dividing the total captured in untreated arms by the total captured in untreated arms and expressing the result as a percentage. For comparisons, the mean percentage deterrent per treatment with a 95% confidence interval was employed. Personal protection was measured as the difference between total blood fed in untreated and total blood fed in treated divided by total blood fed in untreated. The key measure was the mean percentage of personal protection against *An. gambiae* bites per treatment with a 95% confidence interval.

### Statistical analysis

For this experimental hut trial, a power calculation for generalized linear mixed effects models (GLMMs) was undertaken to determine if Pirikool® 300CS (Investigational item) is not inferior than Actellic® 300CS (Active comparator) in generating mosquito mortality.

The logistic regression using R software was used to examine the proportional outcomes (blood feeding and mortality) associated with each experimental hut treatment. In the experimental hut trial, the count results (entrance and exit) were analyzed using Poisson or negative binomial regression. For each result, a different model was fitted. Each model includes random variables to account for sources of variance such as sleepers, as well as fixed factors that were accounted for across treatment and month. The bioassay data was plotted to indicate mortality. The major criteria for assessing IRS in experimental huts were 24-h mortality in the research site's prominent malaria vectors.

### Ethical considerations

The National Ethics Committee for Health and Life Sciences of Côte d'Ivoire (CNESVS) approved the study before it began (N/ref:188-/MSHPCMU/CNESVS-km). Before participating, all volunteers/sleepers provided written informed permission. They received malaria-related chemoprophylaxis and were evaluated daily for evidence of fever or potential treatment adverse effects. A local doctor treated volunteers/sleepers with confirmed *Plasmodium falciparum* infection. The study team interviewed volunteer/sleepers in the huts on a daily basis about the perceived unfavourable or good impacts of the treatments.

## Results

### Experimental hut results

#### Residual efficacy of IRS treatments

**Fig. 1:**
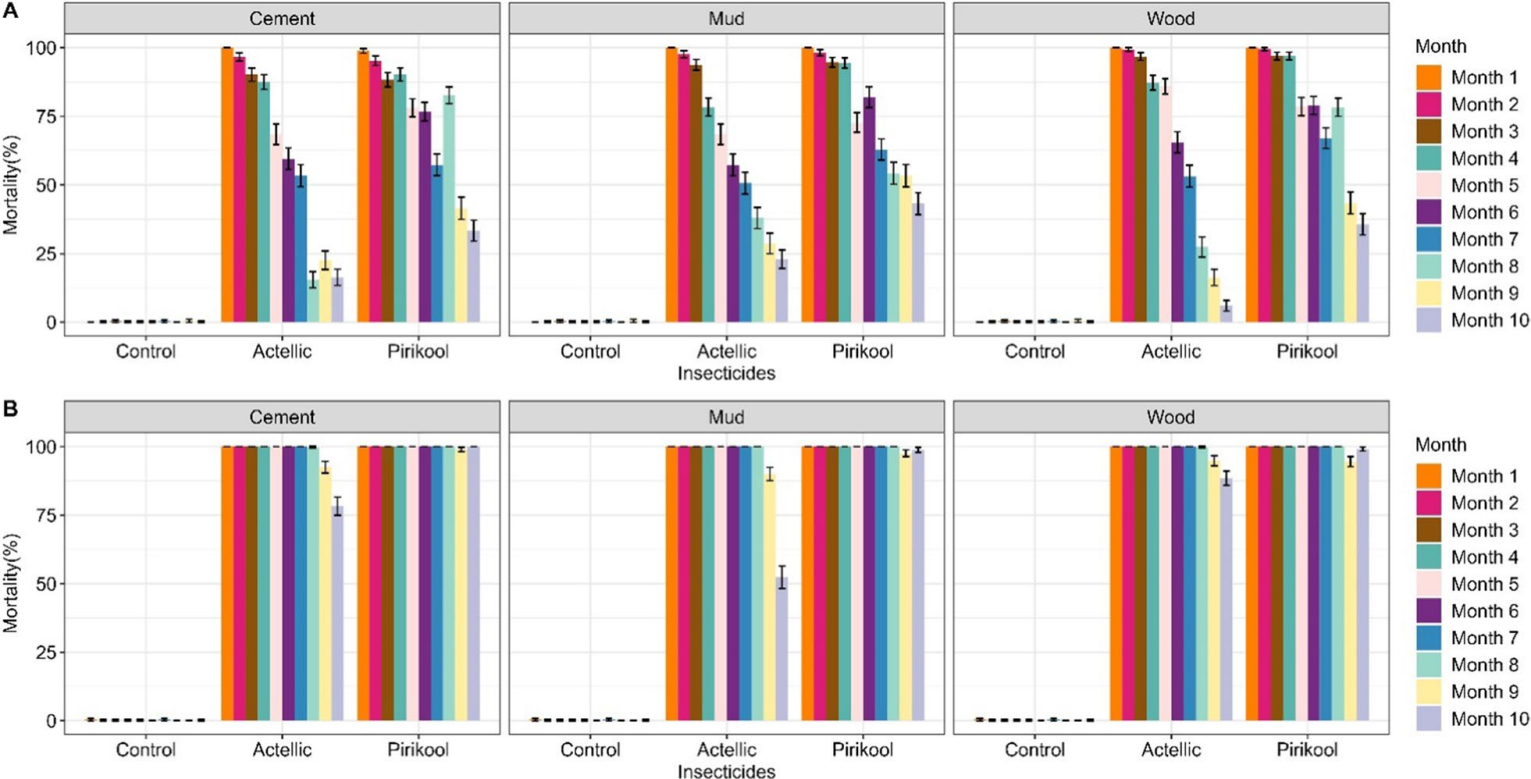
Cone bioassays mortality (24 h) with Tiassalé wild *An. gambiae s.l.* (**A**) and susceptible Kisumu *An. gambiae* s.l. (**B**) on IRS-treated experimental huts

During the first four months, the mortality rate of *An. gambiae* Tiassalé wild resistant strain was 100% for both Actellic® 300CS and Pirikool® 300CS treated substrates and remained above 80% on all Actellic® 300CS treated substrates until the sixth month. Mortality remained over 80% in Pirikool® 300CS treated substrates until the 7th month on mud wall huts and the 8th month on cement and wood wall huts (Fig. 1A). Mortality in cone bioassays with both Actellic® 300CS and Pirikool® 300CS on the three substrates was over 80% and lasted 10 months for the susceptible laboratory *An. gambiae* Kisumu strain (Fig. 1B).

#### Mosquito overall entry and exiting

**Fig. 2:**
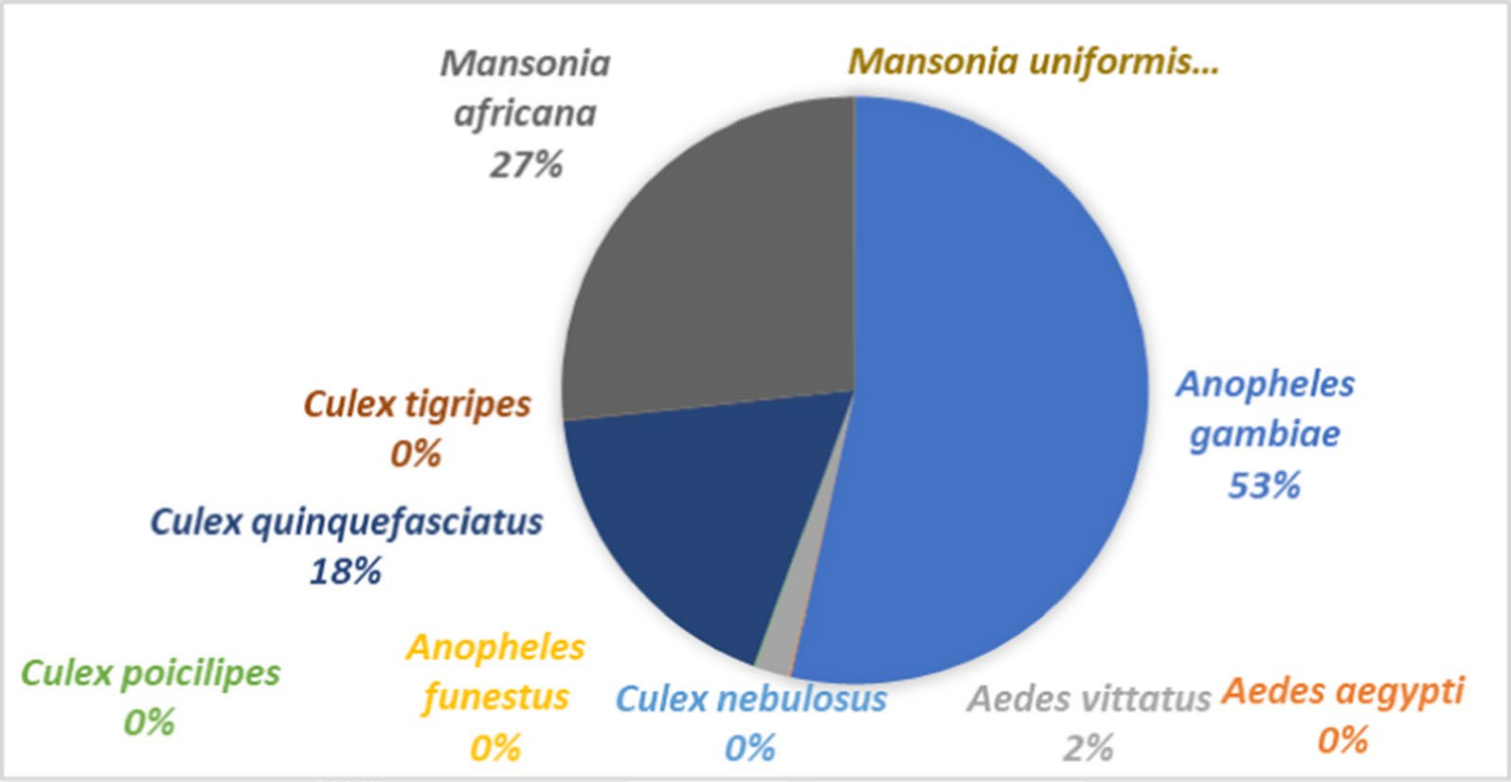
Mosquitoes species collected in Tiassalé experimental huts

A total of 20,505 wild female mosquitos were gathered throughout an eight-month period. *An*. *gambiae* was the most common of these species, accounting for 53%, followed by *Mansonia africana* (27%), *Culex quinquefasciatus* (18%), and *Aedes vittatus* (2%). Other species accounted for less than 1% (Fig. 2). Only *An. gambiae* was subjected to statistical analysis.

#### Deterrent effect

The deterrence observed in cement Actellic® 300CS treated huts (81%) was similar to that observed in cement Pirikool® 300CS treated huts (82.2%; P = 0.35). In mud treated huts, the deterrence was 64.2% and 61.8%, respectively (P = 0.05, Table 1).

**Table 1:** Mean percent of outcomes metric on wild *An. gambiae* s.l. from experimental huts

Products	Substrate	Total collected	Females in veranda	Female blood-fed	Deterrence (%)	Exophiliy (%)	Induced exophily (%)	Blood feed (%)	Personal protection (%)
Water	Cement	3931	1359	2464	NA	34.6 (33.1–36.1)	NA	62.7 (61.2–64.2)	0.0 (0.0–0.2)
Actellic® 300 CS	Cement	1376	691	566	81 (78.9–83.1)	50.2 (47.6–52.9)	15.6 (12.5–18.8)	41.1 (38.6–43.8)	77.0 (75.3–78.6)
Pirikool® 300 CS	Cement	1243	676	476	82.2 (80.5–83.9)	54.4 (51.6–57.1)	15.9 (12.7–19.2)	38.3 (35.6–41.0)	80.7 (79.1–82.2)
Actellic® 300 CS	Mud	1098	373	736	64.2 (60.6–67.8)	34.0 (31.2–36.8)	7.3 (5–9.5)	67.0 (64.2–69.7)	70.2 (68.4–72.0)
Pirikool® 300 CS	Mud	1365	531	805	61.8 (58.1–65.5)	38.9 (36.3–41.5)	11.4 (8.7–14.2)	59.0 (56.3–61.6)	67.4 (65.5–69.2)
Actellic® 300 CS	Wood	1057	482	513	83 (81.2–84.8)	45.6 (42.6–48.6)	10.6 (7.9–13.3)	48.5 (45.5–51.5)	79.2 (77.6–80.8)
Pirikool® 300 CS	Wood	909	319	384	73.5 (70.1–76.9)	35.1 (32.1–38.3)	6.3 (4.1–8.4)	42.2 (39.1–45.5)	84.4 (82.9–85.8)

#### Exit rate

**Fig. 3:**
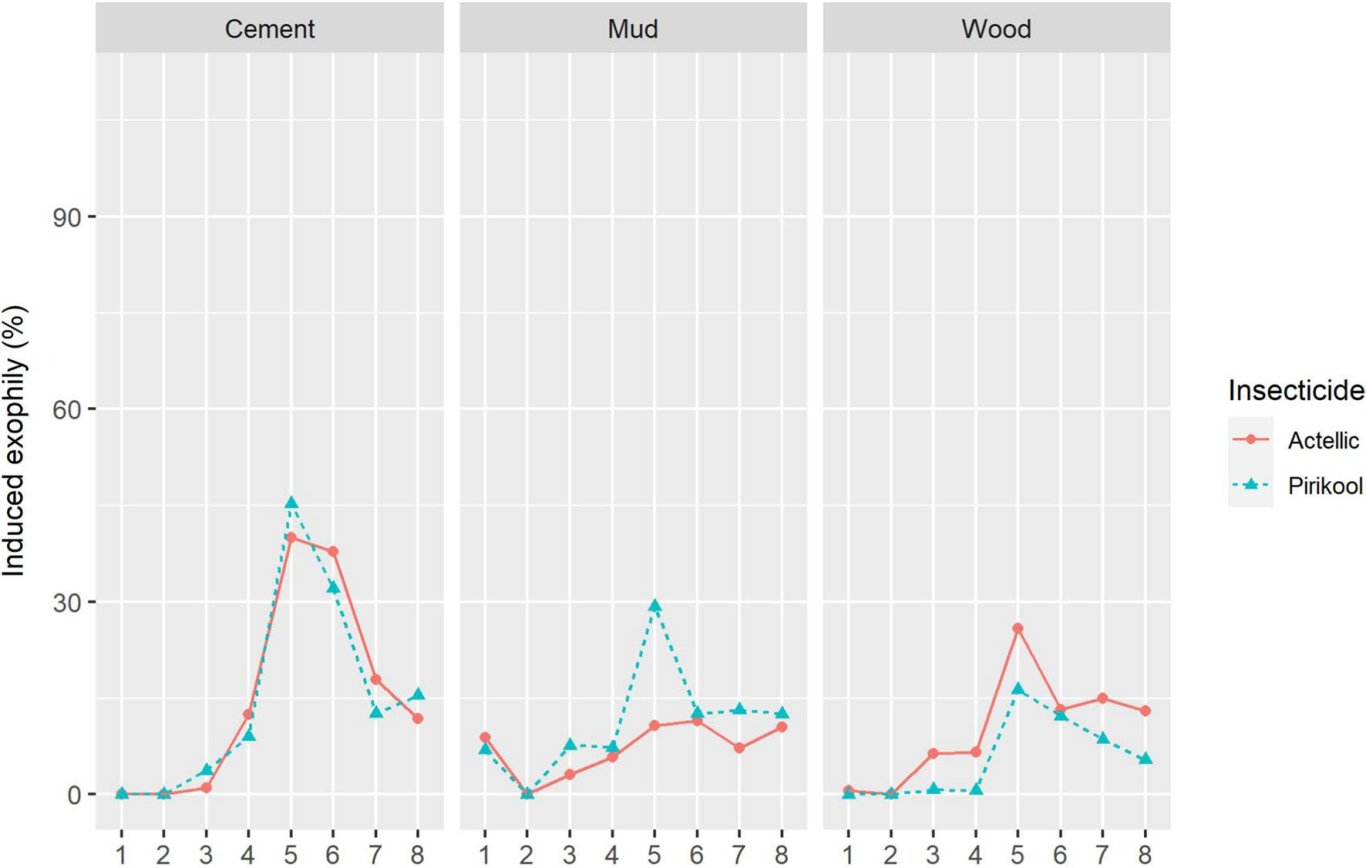
Induced exophily by substrate from Actellic® 300 CS and Pirikool® 300 CS treated huts

The cement wall exit rate did not differ between Actellic® 300CS treated huts (50.2%) and Pirikool® 300CS treated huts (54.5%; P = 0.94). This trend has been noticed in wood-treated huts. Indeed, the exit rate was statistically similar (P > 0.05%) between Actellic® 300CS treated huts (45.6%) and Pirikool® 300CS treated huts (35.1%). However, as shown in Fig. 3, the exit rate was higher with Pirikool® 300CS (38.9%) than Actellic® 300CS (34%; P < 0.05) in mud treated hut.

### Overall blood feeding rates

#### Mortality rates of wild free-flying *An. gambiae* analysis

##### Cement walled huts

**Fig. 4:**
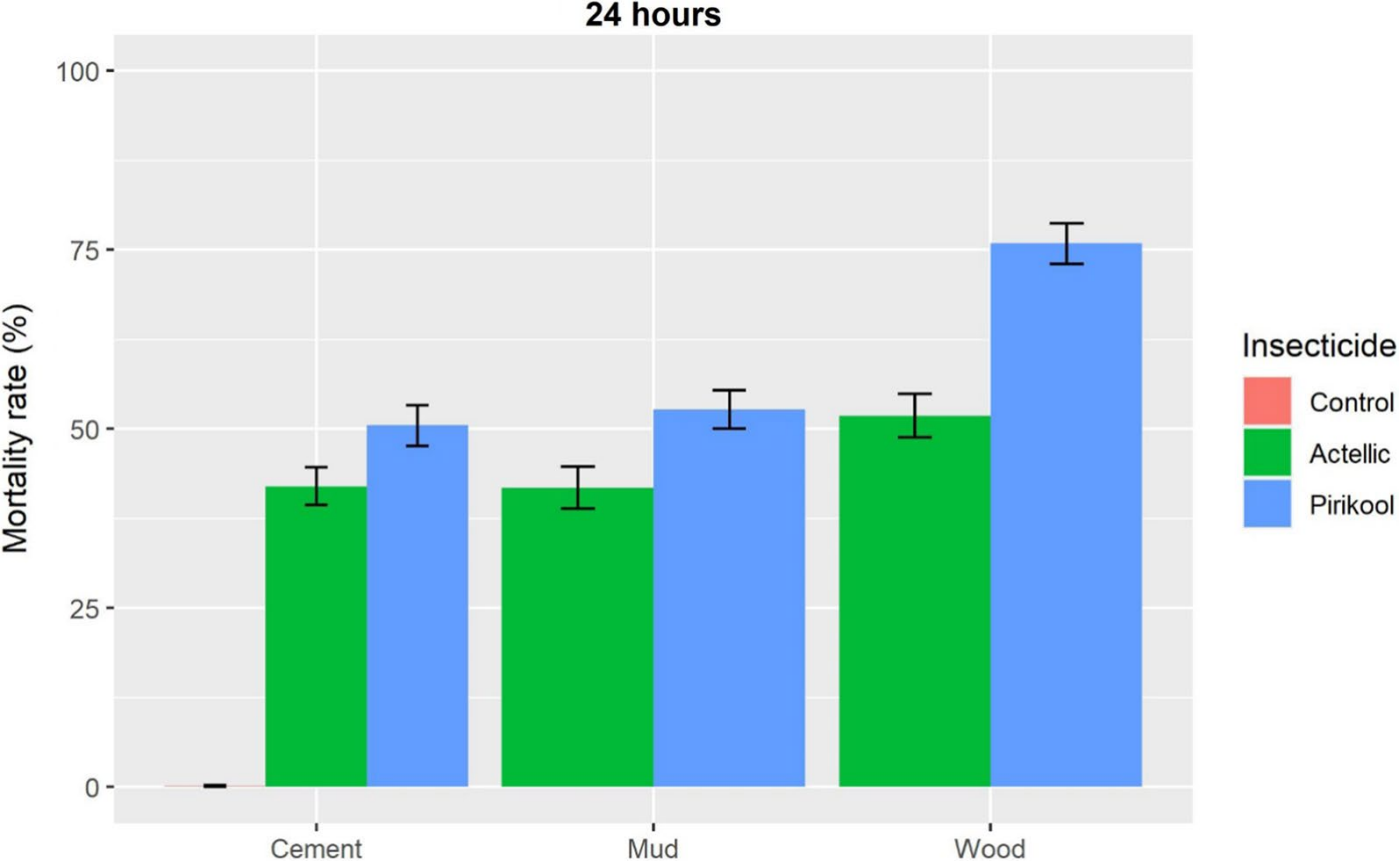
Overall 8-month mean percent mortality rates by substrate of wild free-flying *An. gambiae* s.l

**Fig. 5:**
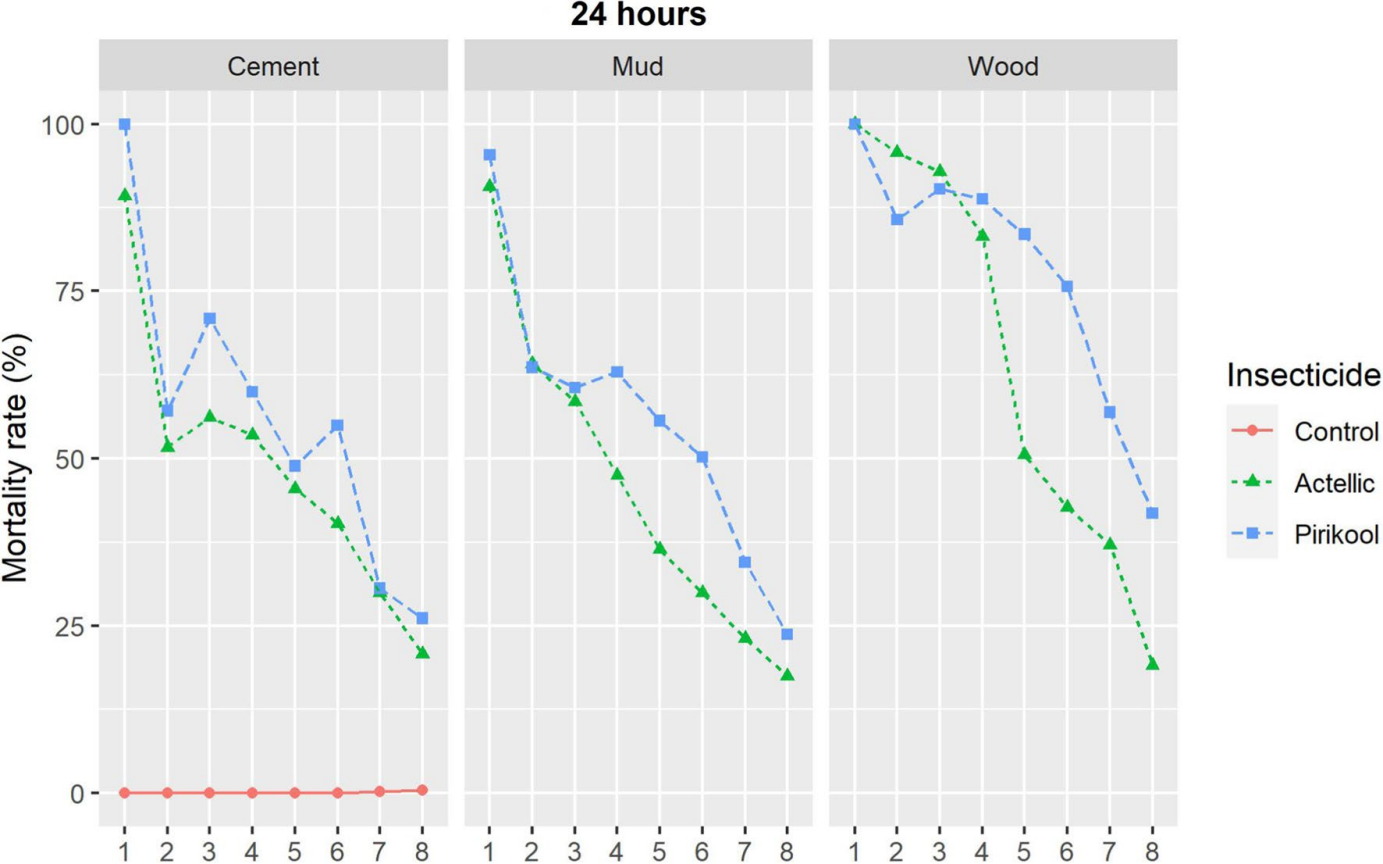
Monthly rates of wild free-flying resistant *An. gambiae* s.l. entering IRS-treated experimental huts from month 1 to month 8

In the first month, mortality on cement-walled huts was 89.2% in Actellic® 300 CS treatments and 100% in Pirikool® 300 CS treatments (Fig. 4). Mortality dropped to 20.8% in Actellic-treated huts and 26.1% in Pirikool-treated huts after eight months (Fig. 5). Logistic regression model 24 h death rates of *An. gambiae* over an 8-month period using WHO criteria of non-inferiority (> 0.7 lower confidence interval) revealed that Pirikool® 300 CS treatments (50.5%) were not inferior to Actellic® 300CS treatments (42.0%; OR = 1.5, 95% CI 0.76–2.97).

##### Wood walled huts

In the first month, both Actellic® 300CS and Pirikool® 300CS treatments resulted in 100% mortality. Eight months later, mortality in the Actellic® 300CS treatments group was lower (19.1%) than in the Pirikool® 300CS treatment group (41.8%). Overall 24 h mortality was 75.9% with Pirikool® 300CS and 51.8% with Actellic® 300CS. Pirikool® 300CS treatments did not result in a lower mortality rate than Actellic® 300CS treatment (OR = 2.52, 95% CI 1.5–4.22).

##### Mud walled huts

Mortality in the first month was slightly higher in the Pirikool® 300CS treatment (95.4%) than in the Actellic® 300CS treatment (90.6%) in the mud walled hut. The mortality rates for Actellic® 300CS and Pirikool® 300CS treatment arms after 8 months were 17.5% and 23.7%, respectively. Pirikool® 300CS showed a greater overall mortality rate (52.7%) than Actellic® 300CS (41.8%), but was not inferior (OR = 1.59, 95% CI 1.31–1.91).

#### Quality of spray application

According to the findings of the HPLC analysis of filter papers used in experimental hut walls after spray treatments with Pirikool® 300CS and Actellic® 300CS given in Table 2, only one hut falls beyond the range of 50% of the target dosages according to the WHO spray quality statement [27].

**Table 2:** Results from HPLC analysis of filter papers from treated experimental huts

	Hut treatments	Replicate	Target dose (mg/m^2^)	Filter paper (mg/m^2^)	Deviation from target dose (%)
Control	Cement	1	0	0	/
		2	0	0	/
		3	0	0	/
Actellic® 300CS	Cement	1	1000	645.1	− 35.5
		2	1000	736.3	− 26.4
		3	1000	895.5	− 10.5
	Mud	1	1000	727.7	− 27.2
		2	1000	1043.8	4.4
		3	1000	707.1	− 29.3
	Wood	1	1000	711.5	− 28.9
		2	1000	622.4	− 37.8
		3	1000	953.7	− 4.6
Pirikool ® 300CS	Cement	1	1000	720.2	− 28.0
		2	1000	1099.3	9.9
		3	1000	1490.7	49.1
	Mud	1	1000	1401.1	40.1
		2	1000	1608.3	60.8
		3	1000	876.4	− 12.4
	Wood	1	1000	946.6	− 5.3
		2	1000	1134.5	13.5
		3	1000	1352	35.2

## Discussion

Evaluations in semi-field and field circumstances are used to choose the most effective pesticides for vector control. The findings of these research are based on an examination of entomological indicators used to assess the efficiency of insecticides on mosquitoes.

Thus, the entomological performance of Pirikool® 300 CS for IRS was determined in this investigation done in experimental huts. An assessment of mosquito populations caught in the experimental huts indicated that the culicidal fauna is extensive and diversified, with *An. gambiae* clearly outnumbering other mosquito species. The imbalance in species abundance is considered to be connected to agricultural methods that promote *An. gambiae* growth. [28]. Indeed, Tiassalé is a rice-producing location thanks to a large, regularly watered area separated into compartments that guarantees rice production throughout the year. According to previous research in Côte d'Ivoire, the dominance of *An. gambiae* would be attributable to the abundance of breeding site provided by flooded and sunlit pits [29].

The findings of this investigation demonstrated a good deterrence of huts sprayed with both Pirikool® 300 CS and Actellic® 300CS, indicating that both treatments perform well against highly resistant *An. gambiae* populations. In general, when an insecticide may induce deterrence, it usually acts to limit human/vector interaction, and so non-users may obtain some indirect protection against mosquito bites in communities where not everyone has access to chemical protection. [30]. However, if the coverage rate of an intervention (ITNs or IRS) is not total at the village level, the deterrent effect at the entrance to the households will have the disadvantage of reducing the proportion of mosquitoes entering in treated houses, allowing untreated houses to receive more mosquitoes than usual. This suggests that 100% coverage of households with vector control methods would give more effective vector protection.

Furthermore, in specific instances, the treatment demonstrated very significant exophily of up to 50%. The great efficiency might be attributed to the application used for this evaluation. Indeed, in the investigation, a broad treatment that included walls and ceilings was used. Thus, mosquitoes entering huts, where the entirety of the interior has been treated, are forced to leave as quickly as possible to avoid contact with the insecticide.

Indeed, research in Soumousso, Burkina Faso [31] found that full application of insecticides in the huts, including wall and ceiling, yielded better outcomes than selective application.

Blood-feeding rates are a crucial statistic indicator to examine when evaluating an insecticide's effectiveness since vectors spread disease infections during blood feeding. A high inhibition to feeding might thus be regarded as the potential insecticide's good performance. However, in this current investigation, feeding inhibition was minimal. In general, vector control attempts to prevent human/vector interaction by reducing bites or eliminating mosquitos that try to feed.

However, according to IRS ideas, vectors would often feed on the host prior to coming into touch with the insecticide, resulting in a high feeding rate, as shown throughout various evaluations [11, 18, 32, 33].

Despite the Tiassalé vectors' insecticide tolerance, Pirikool® 300 CS demonstrated an excellent lethal impact with all three types of substrates. The mortality rates that occurred were equivalent to those achieved with the same substance in a similar research in Benin [18]. The mortality rate generated on wild mosquitos entering in the huts was higher on the wooden hut substrates than with the other two substrate types. These findings contrast those obtained in Covè, Benin, during a study of the effectiveness of clothianidin alone or in combination with deltamethrin in experimental huts. [33]. Indeed, the findings of their investigation revealed that mortality in cement and mud huts were greater than in wooden huts. Given the scarcity of data from research on wooden substrates in experimental huts, it is difficult to determine which type of substrate gives longer-lasting insecticide effectiveness.

In an epidemiological understanding, the mortality induced by an insecticide is a very important factor considered in the evaluation of a product's efficacy because it allows not only the limitation of host/vector contact but also the reduction of longevity and density on vectors, whereas other parameters are limited to the reduction of human/vector contact. Given Pirikool® 300 CS's strong lethal efficacy on vectors, it might be a potential product that could lawfully supplement the products used in IRS in the context of controlling vector resistance to insecticides. Furthermore, a WHO cone bioassay residual utilizing the susceptible *An. gambiae* Kisumu strain performed on the treated experimental hut wall surfaces resulted in mortality rates greater than 80% for a period of 10 months. According to these findings based on WHO standards [4], Pirikool® 300CS used as indoor spraying is effective even in areas where *An. gambiae* is resistant to all four classes of public health insecticides, as observed throughout the research [20, 21, 23].

A recently published study in Côte d'Ivoire found that pirimiphos methyl is one of the rare insecticides that still has high activity against malaria vectors [34]. Though not unexpected, this study demonstrated that Pirikool® 300 CS, a pirimiphos-methyl formulation, worked effectively on resistant vectors in Tiassalé. Negative cross-resistance may explain pyrimiphos-methyl susceptibility. According to a Tiassalé investigation, the overexpression of PY-detoxifying cytochrome P450s in *An. gambiae* in order to withstand pyrethroids makes them sensitive to organophosphates [21].

Pirikool® 300 CS for IRS would be extremely beneficial in malaria intervention programmes in rice-growing areas with a large vector population. However, in terms of resistance management, a combination of Pirikool® 300 CS-based IRS and LLINs in such an area would be more helpful and would assist to boost the effectiveness of each vector control instrument in delaying resistance development.

## Conclusions

The outcomes of this investigation reveal that Pirikool® 300CS has a strong entomological performance in IRS depending on the type of substrate (cement, mud, and wood) against the resistant population of *An. gambiae* in Tiassalé. According to the most recent WHO World Malaria Report, the usage of IRS is obviously dropping, owing mostly to their extremely expensive cost. However, to decrease costs in a good resistance management approach where tools must be combined or alternated, IRS based on a long residual insecticide such as Pirikool ® 300CS may be appropriate.

## Data Availability

Data and materials of this study are included in this article and its additional files.

## Abbreviations

AI: Active ingredient
CS: Capsule suspension
DDT: Dichloro-diphenyl-trichloroethane
HPLC: High performance liquid chromatography
IRS: Indoor residual spray
Kdr: Knockdown resistance
LLIN: Long-lasting insecticidal net
Ace-1: Insensitive acetylcholinesterase
PQT-VCP: Prequalification team for vector control products
WHO: World Health Organization
